# Development and Treatment of Radicular Cyst in Pediatric Patient—Case Report

**DOI:** 10.3390/jcm14020452

**Published:** 2025-01-12

**Authors:** Amadeusz Kuźniarski, Jan Kiryk, Sylwia Kiryk, Edward Kijak, Magdalena Aleksandra Dubowik, Jacek Matys, Maciej Dobrzyński

**Affiliations:** 1 Dental Prosthetic Department, Wroclaw Medical University, Krakowska 26, 50-425 Wroclaw, Poland; amadeusz.kuzniarski@umw.edu.pl (A.K.); edward.kijak@umw.edu.pl (E.K.); 2 Dental Surgery Department, Wroclaw Medical University, Krakowska 26, 50-425 Wroclaw, Poland; jan.kiryk@umw.edu.pl; 3 Department of Pediatric Dentistry and Preclinical Dentistry, Wroclaw Medical University, Krakowska 26, 50-425 Wroclaw, Poland; s.roguzinska@gmail.com (S.K.); maciej.dobrzynski@umw.edu.pl (M.D.); 4 Department of Dentofacial Orthopedics and Orthodontics, Wroclaw Medical University, Krakowska 26, 50-425 Wroclaw, Poland; magdadubowik@gmail.com

**Keywords:** cystotomy, complications, radicular cyst, obturator, pediatric patients, pulpotomy

## Abstract

**Background:** Inflammation in the periapical area of primary teeth can affect the development and eruption of permanent teeth. In an asymptomatic course, they are detected accidentally during routine examinations. In such cases, they often reach significant dimensions and cause irreversible changes. **Methods:** This report presents the case of a 9-year-old boy with a radicular cyst in the mandible, resulting in the displacement of both premolar tooth buds. To address the inflammation, facilitate the proper eruption of the impacted teeth, and prevent adjacent teeth from shifting, the primary molars were extracted. Marsupialization was performed under general anesthesia, followed by the fabrication and placement of an obturator. **Results:** Two months after treatment, the displaced tooth buds began aligning along the natural eruption pathway. By the 8-month follow-up, the premolars had successfully erupted into their proper positions in the oral cavity. **Conclusions:** Marsupialization combined with the use of an obturator is an effective first-line treatment for radicular cysts in pediatric patients, offering a conservative approach that promotes natural tooth eruption while preserving the surrounding dentition.

## 1. Introduction

Dental caries in deciduous teeth affects approximately 50% of the global pediatric population. Some authors have suggested that pulp complications may occur in up to 90% of cases [1,2]. In this case, the choice of treatment method is determined by many factors. In the case of asymptomatic teeth, the choice of treatment method may be either indirect or direct pulp coverage, depending on whether the pulp was exposed during the preparation of the cavity [3]. The success rate for direct pulp capping is 90% to 96%, while for indirect pulp capping, it is approximately 80% [4,5,6]. Another treatment method is pulpotomy. This approach is used when the pulp is exposed with its reversible inflammation, with the presence of healthy tissue in the deeper layers [6,7]. The success rate of this method has been reported to be as high as 82.6% [8]. However, some authors have reported that up to 38% of teeth develop premature root resorption, while chronic pulpitis and internal resorption are also frequently observed complications [4,9]. In rare instances, a root cyst may develop [10].

The World Health Organization (WHO) defines a radicular cyst (RC) as an inflammatory odontogenic cyst associated with the root of a non-vital tooth [11]. Although radicular cysts are the most common jaw cysts, accounting for 60% of all cysts found in this area, the frequency of radicular cysts in primary dentition is approximately 0.5–3.3% [11,12,13]. The majority of radicular cysts in primary teeth are associated with mandibular molars with apical infection caused by caries [12]. They arise from the remnants of the periodontal ligament epithelium as a result of inflammation and the accompanying infiltration of inflammatory cells, which is usually a consequence of pulp necrosis. There seems to be a possible relationship between intracanal drugs used in pulp therapy and characteristic intraepithelial inclusions, which are located in the walls of the cysts and may constitute a site of prolonged antigenic stimulation [12,14,15]. In the initial stage, a radicular cyst usually does not cause any symptoms. However, if left untreated, it can lead to bone distention, bone resorption, delayed tooth eruption, changes in tooth position, mild sensitivity, tooth mobility, and improper development of subsequent teeth in children [10]. The detection of this type of cyst is most often incidental during routine radiographs. They are seen as a round or oblong, well-demarcated, corticated radiolucency communicating with the root of the causative tooth [11]. The differential diagnosis of radicular cysts includes a follicular cyst, Pindborg tumor, periapical cementoma, post-traumatic bone cyst, ameloblastoma, odontogenic keratocyst, and odontogenic fibroma [16]. The diagnosis of a radicular cyst can only be confirmed by performing a histopathological examination. The radicular cyst is lined with a nonkeratinizing squamous epithelium that proliferates and has a characteristic arcade pattern. The wall is composed of inflammatory fibrous tissue, often with foamy histiocytes. Deposits of cholesterol crystals are often seen with foreign body giant cells that may form luminal nodules [11]. The management of a cyst that causes displacement of the tooth buds, when the intention is to retain the affected teeth, involves either complete extraction or decompression in cases of significant lesions in close proximity to sensitive anatomical structures [17,18]. Surgical treatment of cysts involves various techniques, with one of the most commonly used being marsupialization. This technique is preferred for benign cysts because it is less invasive than enucleation and minimizes the risk of complications such as nerve damage, jaw fracture, or sinus perforation. Acrylic resin obturators are particularly indicated in cases where the cyst is large and located near important anatomical structures. In such cases, the use of obturators during marsupialization can prevent complications that may arise during more invasive procedures [19,20].

Prosthetic obturators are widely used in dentistry; mainly, their effectiveness can be indicated in the postoperative rehabilitation of patients after the surgical resection of oral cancers. They improve oral functions such as chewing and speech and help to close palatal defects [21,22]. Prosthetic obturators are also an effective tool in the treatment of cysts, especially in the context of marsupialization. Prosthetic obturators are used to maintain the surgical opening during the healing process, thus ensuring the success of the surgery. The use of acrylic obturators is essential to maintain the surgical opening, which is crucial for optimal healing and hygiene after marsupialization. Treatment of cysts with prosthetic obturators is an effective method of the rehabilitation of patients with oral defects that may arise after the removal of a cyst or tumor. They help maintain the surgical opening, improve hygiene and quality of life of patients—allowing normal functioning—and offer a unique opportunity for the proper development of permanent teeth [20,23]. The prosthetic obturator fulfills three basic functions: it tightly closes the postoperative cavity while allowing for gradual decompression, and it acts as a space maintainer and facilitates the eruption path of the permanent tooth [20,24,25].

The aim of this case report is to demonstrate the successful management of a radicular cyst in an uncooperative pediatric patient using marsupialization combined with a prosthetic obturator, highlighting its role in reducing inflammation, facilitating the proper eruption of impacted teeth, and preserving adjacent dentition, while providing a conservative treatment approach to prevent complications associated with cystic lesions.

## 2. Case Report

**Figure 1:**
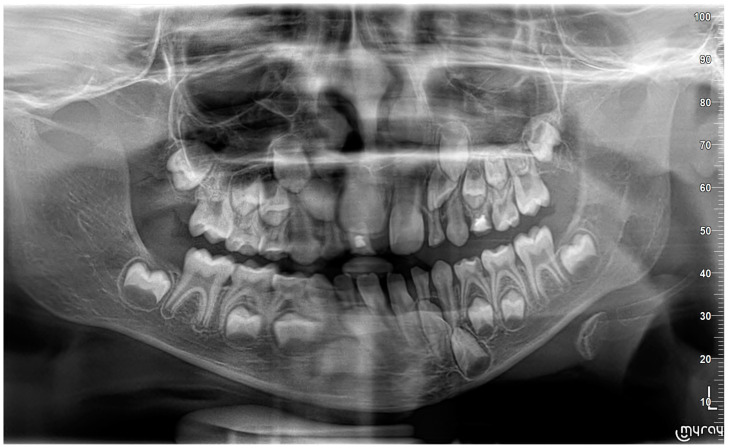
Tooth 74 with a distal defect involving the pulp horn.

**Figure 2:**
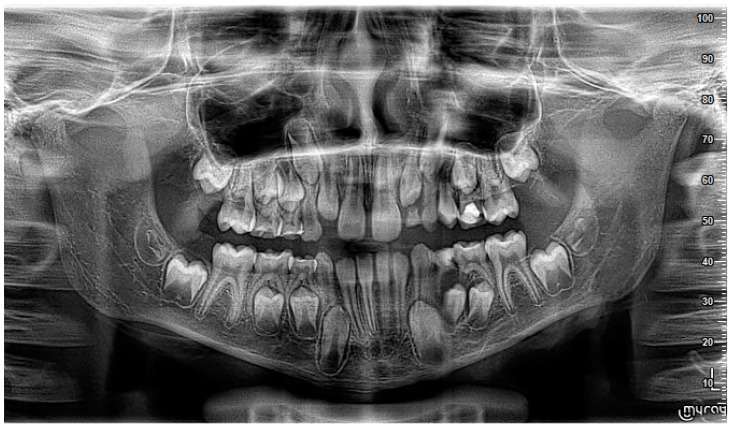
Tooth 74 after amputation treatment 18 months later compared to Figure 1. Slight enlargement of the tooth 34 follicle is visible.

**Figure 3:**
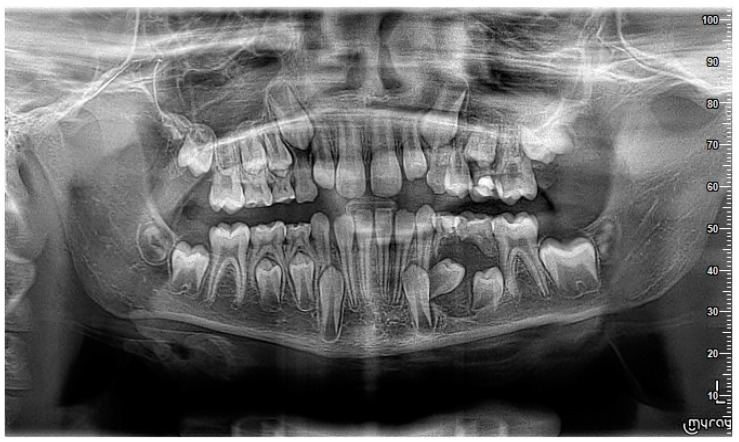
A visible spherical osteolytic lesion of the inflammatory cyst nature at teeth 74 and 75 along with the resorption of the roots of primary teeth. Progression within 6 months to the performance of Figure 2.

**Figure 4:**
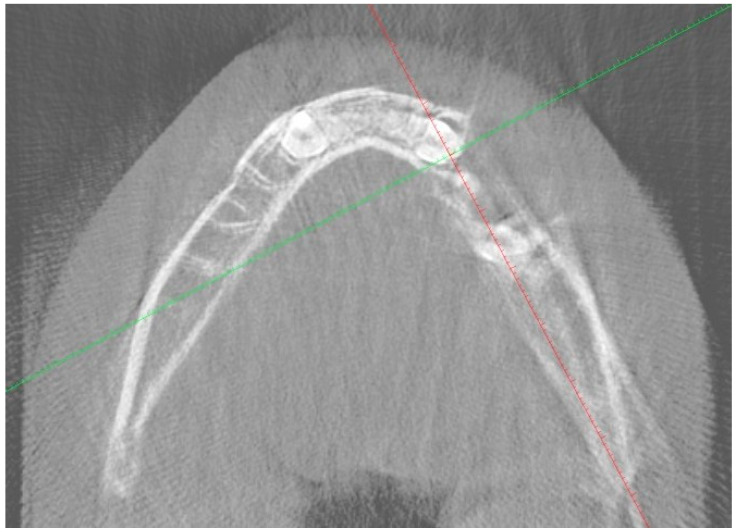
CBCT image in horizontal plane.

**Figure 5:**
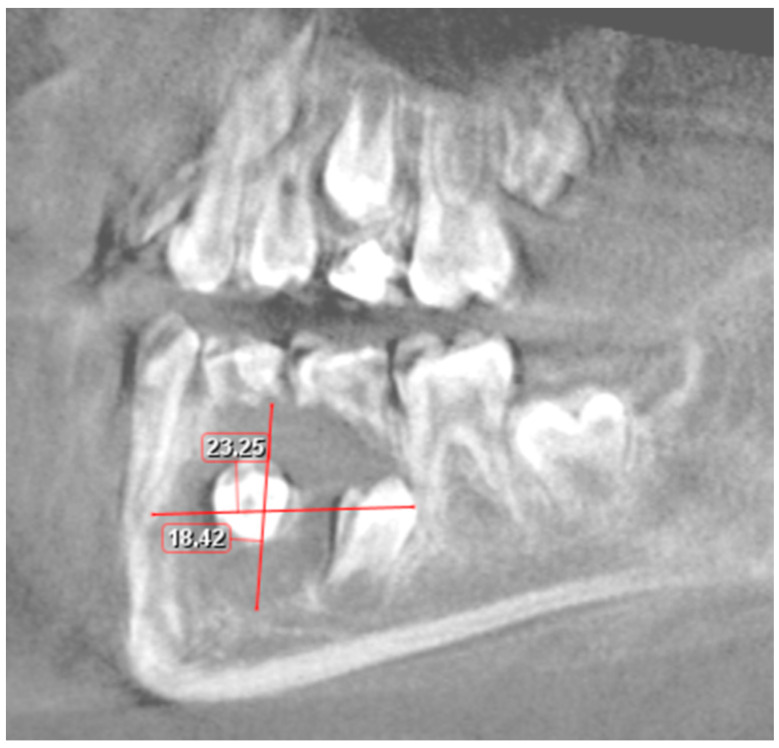
Sagittal CBCT image. Visible cyst measurements.

A nine-year-old male patient was referred to the Department of General Anesthesia at the University Dental Centre in Wroclaw for the management of a periapical lesion associated with primary first and second molars (74 and 75 according to Viohl system [26]). The patient presented with radiological documentation provided by a dental surgeon, which included a series of radiographs (Figure 1, Figure 2 and Figure 3). Initial imaging revealed a cavity in tooth 74, which had undergone coronal pulp amputation. A subsequent radiograph, obtained for orthodontic treatment planning, revealed an enlarged follicle of tooth 34 and the distal root resorption of tooth 74 (Figure 2). A third radiograph, obtained to monitor the amputation treatment of teeth 64 and 74, revealed a spherical osteolytic lesion suggestive of a radicular cyst (Figure 3). The only finding on clinical examination was alveolar process distension. To enhance diagnostic accuracy and assess the extent of the lesion, cone-beam computed tomography (CBCT) was performed (Figure 4 and Figure 5). Due to difficult cooperation with the patient, the CBCT images were slightly blurred, but it was decided that they were sufficient for the diagnosis and in order not to expose the patient to X-ray radiation, the examination was not repeated.

**Figure 6:**
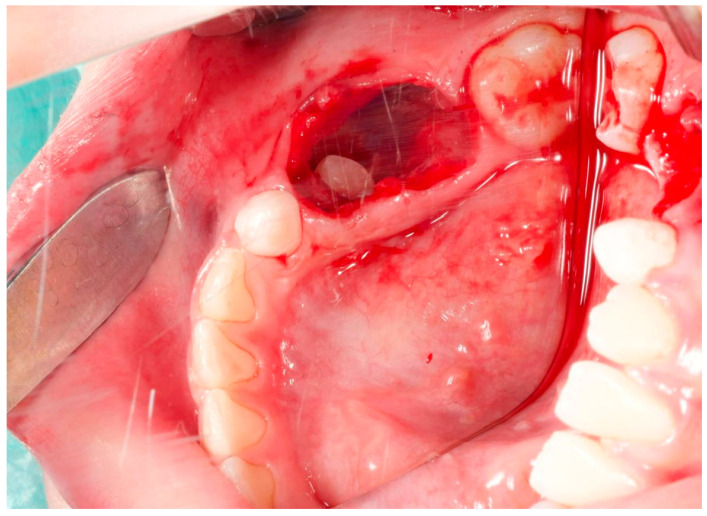
Intraoperative image of cyst cavity.

Due to the complexity of the procedure and the patient’s limited capacity for cooperation, the attending physician determined that general anesthesia was necessary, in alignment with the parents’ wishes. The patient was induced under general anesthesia using 150 mg of propofol; then, after intubating the patient, anesthesia was continued using sevoflurane at a concentration of 2.5–3 vol%. Then, the surgical site was cleansed to minimize the risk of contamination. To achieve hemostasis and reduce intraoperative bleeding and postoperative discomfort, 1.8 mL of 4% articaine with adrenaline at a dilution of 1:100,000 was injected into the operative field. A periotome was used to tear the periprosthetic ligaments, facilitating the extraction of teeth 74 and 75 with forceps. The upper wall of the lesion was carefully excised using a scalpel, and the tissue was sent for histopathological examination for the differential diagnosis and confirmation of the diagnosis. The examination of the cavity revealed the visible permanent teeth buds at its base (Figure 6).

**Figure 7:**
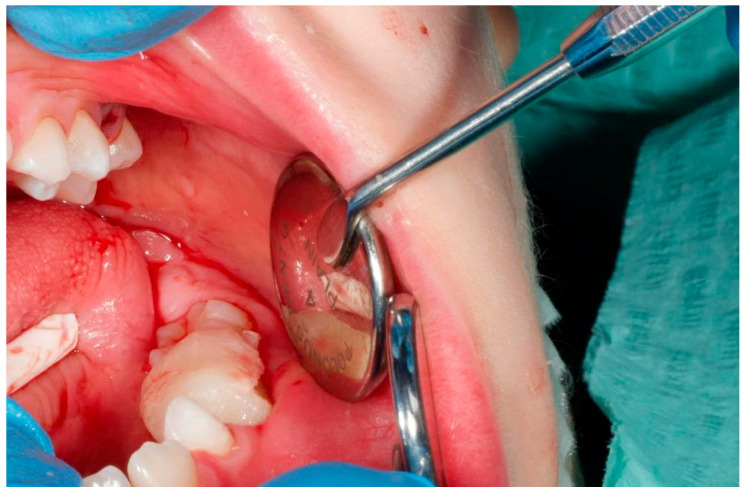
Wax index in cyst cavity.

Due to the location and extent of the lesion, the decision was made to use an acrylic obturator. The surgical wax was applied in the operating area. Bone wax is a sterile mixture of beeswax and isopropyl palmitate, a wax-softening agent, used to help control bleeding from bone surfaces used extensively in dental surgery (Figure 7). Due to its properties (sterility and plasticity), it is ideal for the restoration of prosthetic surfaces. After plasticization, the wax was inserted into the postoperative cavity and shaped according to the rules, i.e., the shaft and cap were formed to ensure tight filling while maintaining stability. The literature often indicates the use of alginate impressions, while based on our clinical experience, the wax method provides more control during the impression taking, mainly without the risk of tearing the alginate impression material [20,27].

After the preparation and verification of the position, size, location in the cyst cavity, and stabilization on the alveolar process, an alginate impression was made to create an obturation plate (with bone wax obturator index). The thermoplastic plate permanently bonded to the fabricated obturator provides additional retention and stability during the period of use. The obturation plate and the obturator will not impede bone development, due to the potentially short lifespan.

**Figure 8:**
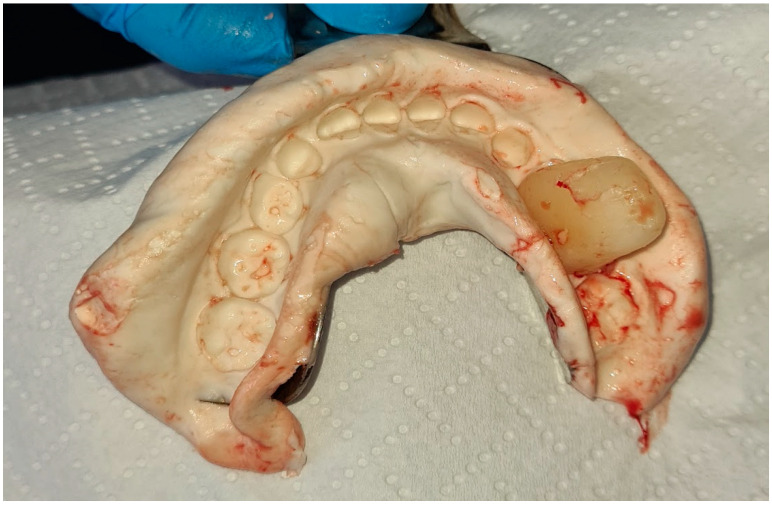
Impression with index.

**Figure 9:**
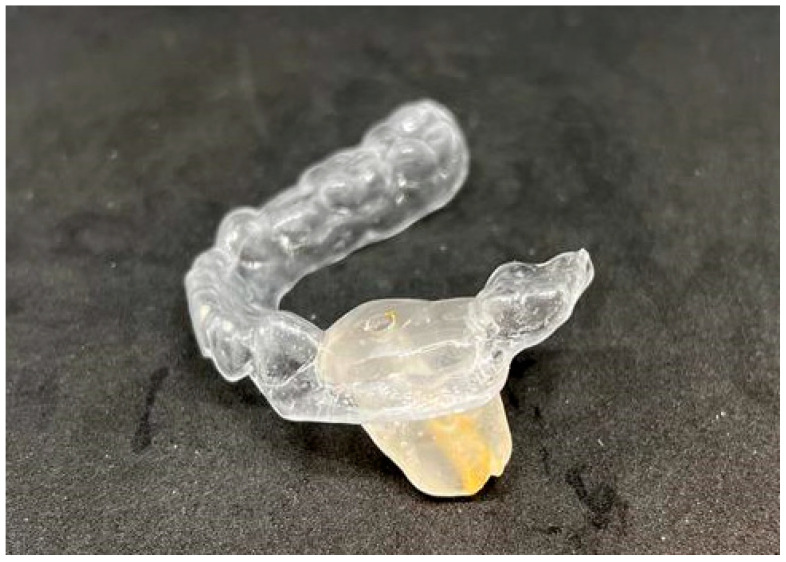
Obturator.

**Figure 10:**
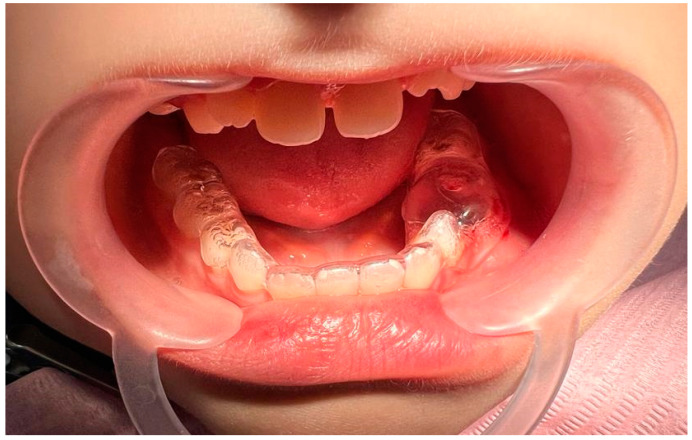
The patient was fitted with an obturator the same day, 3 h after the procedure.

After the completion of the bone wax index and alginate impression, the work was transferred to a technical laboratory (Figure 8). The wound was secured with a seton moistened with a saline solution, which was then sutured to the edges of the wound. Following the patient’s awakening, he was referred to the Prosthodontics Clinic for the continuation of his treatment on the same day (Figure 9 and Figure 10).

## 3. Follow-Up and Outcomes

Follow-up visits for the clinical observation of a patient are crucial in the management of prosthetic treatment. The protocol for patient follow-up visits was as follows: the patient had to be seen once a week for three consecutive weeks, then every two weeks until three to four months had passed. The follow-up visits included the following elements:

(a) Assessment of the degree of healing of the post-resection wound;

(b) Assessment of the degree of tightness of the obturator;

(c) Assessment of the state of hygiene of the dentition and prosthetic restoration;

(d) The visual observation of the position of the permanent teeth;

(e) Comments on the daily use of the prosthetic restoration by both the patient and his parents.

In order to minimize the young patient’s exposure to X-rays, only necessary imaging examinations were performed.

During each follow-up visit, the obturator was systematically adjusted by reducing its circumference and length. These modifications were guided by clinical examination findings, including changes in cyst size and the degree of permanent tooth eruption observed within the postoperative cavity, as well as patient-reported feedback. Such adjustments were essential to facilitate the eruption of permanent teeth while maintaining an appropriate fit. Evidence from the literature indicates that, during follow-up visits, the obturator should be progressively shortened to ensure that it adequately seals the wound without exerting undue pressure on the erupting tooth. Waly’s study reported that this adaptation process can span from 3 to 6 months [28].

**Figure 11:**
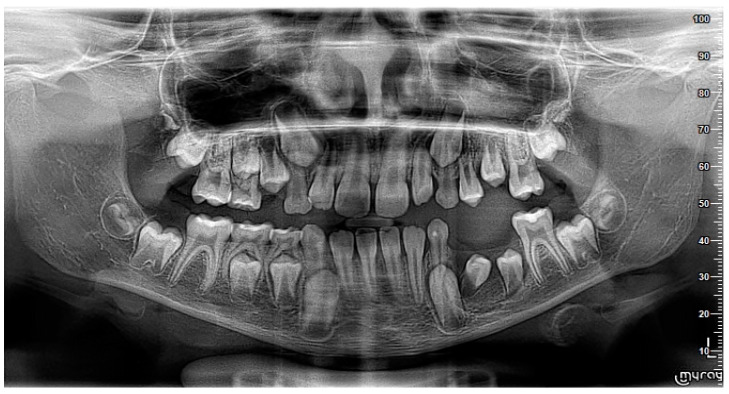
The visible reconstruction of the trabecular bone at the site of the cyst cavity and eruption of teeth 34 and 35. Image: 2 months after treatment.

Two months after the procedure, during radiological control, the restoration of the bone structure and eruption of teeth 34 and 35 were observed (Figure 11). Histopathological examination confirmed the clinical diagnosis of a radicular cyst.

During follow-up visits and subsequent corrections, neither the patient nor his parents reported any problems with the use of the obturator. From our clinical experience and from the literature, it appears that children show exceptional potential for rapid adaptation to dental prostheses [29]. After 3 months of using the obturator, when the erupting permanent teeth had reached the level of the gums, it was decided to discontinue the use of the obturator plate to enable further eruption until occlusal contacts were achieved and the teeth were properly positioned in the dental arch.

**Figure 12:**
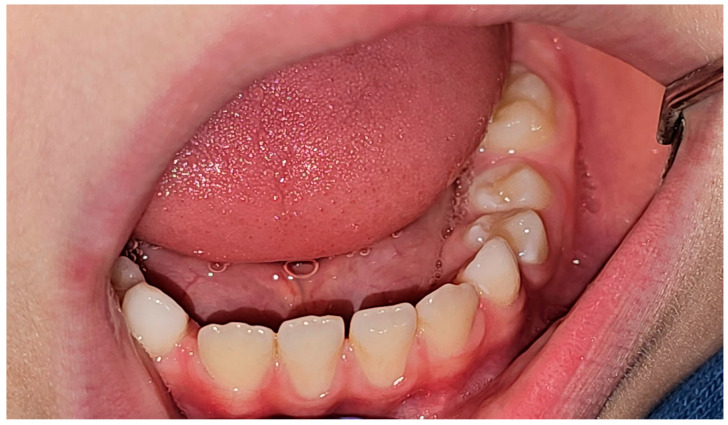
Clinical image 8 months after procedure.

**Figure 13:**
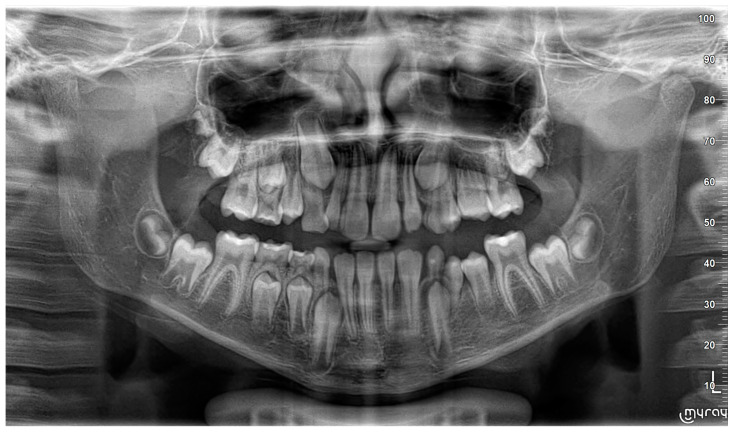
Radiological image 8 months after procedure.

A follow-up examination conducted eight months after surgery revealed that the premolars (34 and 35) were positioned correctly, with no vestibular shallowing or loss of keratinized gingiva. Radiographic imaging showed the complete healing of the lesion and restoration of the bone structure (Figure 12 and Figure 13).

## 4. Discussion

The location of the cyst in the presented case is consistent with the literature. In the study by Mass et al. [12], the primary molars were the teeth most frequently affected by radicular cysts in the primary dentition. Furthermore, according to the study by Manekar et al. [30], the mandible was more frequently affected by primary molar cysts than the maxilla. The literature indicates that there are specific clinical features of radicular cysts developing in primary teeth after pulp treatment: including buccal expansion, large size, and rapid growth [15]. Mass et al. [3] reported 4 out of 36 cases of cysts associated with primary molars after pulp treatment. In the study by Grundy et al. [15], 17 cases received pulp treatment. The only common component of the drugs used was the phenolic group. Grundy et al. considered that continuous antigenic stimulation is caused by drugs used in pulp treatment. This does not mean that it is necessary to prohibit drugs used in root canal treatment of primary teeth, because the occurrence of radicular cysts is very rare. However, teeth after such treatment require constant monitoring. Benn and Altini [31] proposed three possible mechanisms of cyst histogenesis. The first hypothesis suggests that the developmental odontogenic cyst may arise from a dental follicle secondarily inflamed by a dead primary tooth as the source of inflammation. The second proposed explanation would be the formation of a radicular cyst at the apex of a necrotic primary tooth, followed by the eruption of its permanent successor into the radicular cyst, resulting in an odontogenic cyst of extrafollicular origin. They also suggested that the follicle of the permanent successor may become secondarily infected from sources other than the necrotic primary predecessor, leading to the formation of an odontogenic cyst. It is generally accepted that the extraction of a necrotic primary tooth and marsupialization will enable rapid healing of the lesion and eruption of the permanent tooth, provided that these procedures are performed within the normal eruption time [32,33]. By extracting the infected primary teeth, marsupializing the cysts, and providing continuous drainage, it is possible to achieve the spontaneous eruption of the involved permanent teeth, even if they are severely dislocated. The healing and ossification of the bony defect may occur simultaneously with the eruption of the permanent teeth. The repair process is completed within one to two years, depending on the size of the bony defect [34].

In the described case, drainage was maintained with an obturator, which also acted as a space maintainer, prevented food clogging, and allowed access for regular cleaning. The same method proved to be effective in the case presented by Chouchene et al. [35]. There are also reports of the effectiveness of treating similar cases without the use of an obturator [33], although this seems to be more risky due to the possibility of displacement of adjacent teeth, making it difficult for the impacted tooth to erupt properly and contributing to the accumulation of food debris.

The design and construction of the obturator depends on a number of factors, including the location of the resection wound, the number of teeth, and occlusal contacts, which affect the design possibilities of the retention elements. Our experience allows us to distinguish three main groups: stand-alone acrylic obturators with retention clasps or orthodontic brackets, the use of thermoformable splints, and obturators in the form of partial or complete dentures. The third group includes combined obturators, which are designed and manufactured using acrylics. Slim et al. [36] indicate a multitude of therapeutic solutions, such as the use of nasopharyngeal tubes, resin plugs or rubber, resin, silastic, or polyethylene tubes, and the production of obturators from acrylic resin. This indicates a lack of standardized clinical procedures and, in our opinion, requires further investigation and the definition of guidelines. Obturators in the treatment of cyst decompression have many advantages for clinical management. Gurung et al. [24] point out the advantages regarding the use of obturators. These include a reduction in the number of visits, prevention of food-caused infection, and maintenance of proper hygiene at the surgical site.

It is worth noting that Murakami et al. [25] based their study on 100 treatment histories of patients who underwent obturator placement during cyst marsupialization at Kagoshima University Hospital between 31 May 2012 and 31 March 2015. They took into account the patient’s age, gender, remaining teeth, nature of the primary disease, anteroposterior location of the lesion, buccolingual direction of marsupialization, type of obturator, and timing of obturator placement and removal. Based on their findings, they concluded that obturator design had minimal impact on its ability to maintain the surgical opening, with a preference for using the least invasive design. In the case of pediatric patients, especially those treated under general anesthesia, we significantly extend the time of obturation and reduce the risk of patient injury. Ryu et al. [37] additionally indicate that this may cause clinical problems, such as irritation or damage to adjacent tissues, as well as patient discomfort or damage to the obturator due to cyst shrinkage. As a result, it may result in a lack of patient cooperation and non-adherence to recommendations. They point out that also simplifying the decompression technique, e.g., by using a metal tube modified from an intravenous needle, while maintaining appropriate stabilization may end with a successful treatment. Tran et al. [38] emphasize that the cooperation of the patient’s family is also responsible for the success of the treatment. In the process of surgical treatment of cysts with obturators, and due to the age of the patient, it seems reasonable to include orthodontists in the treatment process. Unfortunately, Slim et al. [36] shows that only 1.3% of patients had received orthodontic treatment after marsupialization or decompression. This is an important voice in the discussion on guideline development in line with a holistic approach to treating a patient—especially a minor.

The limitation of the above study is the description of a single case of a patient treated under general anesthesia, which allowed the precise preparation of the prosthetic component. The authors of this paper are currently preparing other cases and are going to prepare a final protocol, taking into account the age, general condition of the patient—including the possible degree of impairment—the location and extent of the cystic lesion, and the presence of other teeth, which have a critical impact on the retention and tightness of the proposed obturator. Despite this, future studies exploring various methods of obturator fabrication in similar cases are necessary.

## 5. Conclusions

The use of obturators in the treatment of large cystic lesions in children

- Reduces the scope of the surgical procedure;
- May help in the proper development of diseased permanent tooth buds;
- May enable proper bone reconstruction;
- Enables the further proper development of occlusion.

Therefore, in the authors’ opinion, it is worth considering the introduction of such a treatment protocol. The size of cystic lesions, their location, the possibility of making an obturator, cooperation with the patient and their caregivers, and the possibility of follow-up visits will determine the success of the treatment.

## Data Availability

Availability of supporting data—The datasets used and/or analyzed during the current study are available from the corresponding author on reasonable request.
